# The Role of the Organization in Promoting Information Security–Related Behavior Among Resident Physicians in Hospitals in Germany: Cross-Sectional Questionnaire Study

**DOI:** 10.2196/46257

**Published:** 2025-01-07

**Authors:** Judith Kraushaar, Sabine Bohnet-Joschko

**Affiliations:** 1 Chair of Healthcare Management and Innovation Faculty of Management, Economics and Society Witten/Herdecke University Witten Germany

**Keywords:** information security, compliance, work engagement, awareness, leadership, communication, education and training, security, privacy, structural equation modeling, resident, fellow, medical education, continuing education, professional development

## Abstract

**Background:**

Nowadays, optimal patient care should be based on data-driven decisions. In the course of digitization, hospitals, in particular, are becoming complex organizations with an enormously high density of digital information. Ensuring information security is, therefore, essential and has become a major challenge. Researchers have shown that—in addition to technological and regulatory measures—it is also necessary for all employees to follow security policies and consciously use information technology (compliance), because noncompliance can lead to security breaches with far-reaching consequences for the organization. There is little empirical research on information security–related behavior in hospitals and its organizational antecedents.

**Objective:**

This study aimed to explore the impact of specific job demands and resources on resident physicians’ information security–related compliance in hospitals through the mediating role of work engagement and information security–related awareness.

**Methods:**

We used a cross-sectional, survey-based study design to collect relevant data from our target population, namely resident physicians in hospitals. For data analysis, we applied structural equation modeling. Our research model consisted of a total of 7 job demands and resources as exogenous variables, 2 mediators, and information security–related compliance as the endogenous variable.

**Results:**

Overall, data from 281 participating physicians were included in the analyses. Both mediators—work engagement and awareness—had a significant positive effect on information security–related compliance (β=.208*, P*=.001 vs β=.552, *P*<.001). Quality of leadership was found to be the only resource with a significant indirect effect on physicians’ compliance, mediated by work engagement (β=.086, *P*=.03). Furthermore, awareness mediated the relationships between information security–related communication and information security–related compliance (β=.192, *P*<.001), as well as between further education and training and the endogenous variable (β=.096, *P*=.02). Contrary to our hypothesis, IT resources had a negative effect on compliance, mediated by awareness (β=–.114, *P*=.02).

**Conclusions:**

This study provides new insights into how a high standard of information security compliance among resident physicians could be achieved through strengthening physicians’ security work engagement and awareness. Hospital management is required to establish an information security culture that is informative and motivating and that raises awareness. Particular attention should be paid to the quality of leadership, further education and training, as well as clear communication.

## Introduction

### Information Security–Related Issues in Hospitals

Nowadays, direct, easy, and quick access to and transmission of information are essential factors for health professionals to provide optimal patient care based on data-driven diagnosis and treatment. In the course of digitization, hospitals, in particular, are becoming complex organizations with an enormously high density of such information, which comes from a wide variety of sources and is available in different information systems. In order to make the decision-making process in hospitals more efficient, increasingly more (medical) devices are being digitally connected to hospital networks, providing health care professionals with data from multiple sources at a central access point. In a recently published study, the average number of active medical devices in large university hospitals in Germany was 25,150, with 4500 (17.9%) of them being digitally connected, a rate that is expected to increase rapidly in the near future [1]. Despite their major benefits in terms of the enhanced quality and efficiency of patient care, the use and high variety of connected devices poses potential information security risks to hospitals. Many of the devices used in hospitals are not only connected to local networks but also directly or indirectly connected to the internet or other external systems. For example, patient data can be transitioned to other stakeholders outside the local network or a cloud where they will be further processed [2,3]. Adding to this, with the increasing usage of personal mobile devices for work purposes by health care professionals, the issue becomes even more complex and difficult to handle for hospital management [4-6].

Information security in hospitals refers to the state of full functionality of all information systems, processes, and components and, therefore, the protection of all information required for optimal patient care. It must be guaranteed at all times. Continuous monitoring, as well as rapid responses to breaches and attacks, are essential. Due to the immense impact of poor information security on patient care, as well as the critical standard of security due to years of underinvestment and neglect of this issue, hospitals around the world have recently become targets of cyberattacks, with massive increases each year [7-10]. A study from 2020 shows how vulnerable the German hospital landscape is to ransomware attacks: the authors analyzed the publicly visible system attack surface of hospitals located in Germany and found that more than 1 in 3 hospitals analyzed had vulnerabilities, including those that were part of the critical infrastructure at the time [11]. In times of scarce financial and human resources, ensuring information security is an enormous challenge [4]. In addition to technological and regulatory measures, research literature also highlights and discusses organizational measures to enhance information security in hospitals [2,8,9]. The reason for this is that a large proportion of information security breaches still occur internally as a result of employee misconduct [2,9,12]. German politicians have already recognized that a high level of information security in hospitals can only be achieved with holistic measures at a human, technological, and organizational level. In addition to providing the technological basis, employees must be made aware of security-relevant issues and obliged and motivated to comply with policies. The hospital management must revise information and communication processes and create appropriate support structures (eg, responsibilities, IT, and training) [13].

Empirical research on information security–related behavior of employees at work and its supporting organizational and individual factors is a relatively new field. Several researchers have found that in addition to the technological equipment of the organization to ensure information security, it is also necessary for the employees to follow security policies and consciously use information technology (compliance), because noncompliance can lead to security breaches with far-reaching consequences for the organization.

To explain information security–related behavior within the organization, various behavioral theories and frameworks have been used, including the Theory of Planned Behavior (TPB), Organizational Behavior (OB), and the Job Demands-Resources Model (JDRM) [14]. Hu et al [15] used the TPB to investigate the role of top management, organizational culture, and individual cognitive beliefs on information security–related behavior among alumni of Management Information Systems and Master of Business Administration programs, finding significant effects. D’Arcy and Greene [16] combined different theories for their research model and examined the influence of security culture, job satisfaction, and perceived organizational support on security compliance intentions among computer-using professionals, finding positive effects on security culture and job satisfaction. Solomon and Brown [17] used the TPB and showed relationships between organizational culture, information security culture—as an organizational subculture—and compliance. They further argued that goal orientation among employees has a stronger influence on compliance than rule orientation.

There is little empirical research on information security behavior in hospitals. Yeng et al [18] recently conducted a study in a paperless hospital in Ghana and assessed the security behavior of health care staff. In their study, the authors covered different individual, psychosocial, cultural, and work factors and found several significant correlations with participants’ security behavior [18]. In their case studies, Hedström et al [19] showed that employees in health care organizations are exposed to different value conflicts, for example, health care values versus information security values, which they have to resolve quickly for each situation during their practice. This poses security risks that need to be considered by hospital management [19]. In our first article on information security published in 2022, we examined specific security-related practices of physicians when using smartphones and medical apps in everyday clinical practice. Among the apps examined, some were found to pose a certain risk to data protection, information security, and patient safety. A large majority of the participating physicians who communicated by their smartphone did not use General Data Protection Regulation (GDPR)–compliant messenger services and paid little attention to some security-relevant criteria (such as manufacturer information, information on data protection, and information security) when selecting medical apps for professional use [20]. Both the papers from 2022 described above and the following paper are based on the same dataset. While our first paper focused on specific security-related practices, this paper builds on this and examines the underlying constructs of this behavior.

Due to the particular threat of cyberattacks, the ongoing digitization, increasing regulatory requirements and the existing research gap on information security–related behavior in hospitals, the aim of this study was to explore the impact of job demands and job resources on resident physicians’ information security–related compliance.

### Theoretical Framework

To develop our research model, we used elements from the Extended JDRM as well as the OB research field.

#### JDRM Framework

The JDRM by Demerouti et al [21] postulates that working conditions can be divided into 2 categories—demands and resources—and are associated with different job-related outcomes. Demerouti et al [21] originally intended to use the JDRM to explain the development of burnout: in one of their studies, they confirmed the links between high demands and exhaustion and between a lack of resources and disengagement [21]. Three years later, a revised version was presented by Schaufeli and Bakker [22]. Their model included burnout and engagement as mediators between different predictors and different possible consequences. Since then, the JDRM has served as a theoretical basis in many studies in industrial and organizational psychology [23]. The JDRM has proven to be flexible, with researchers adapting it to their specific study context regarding the outcomes, demands, and resources to be considered, as well as the presumed mediators and moderators [24]. Pham et al [25] used the JDRM for their qualitative research on the information security–related behavior of employees in Vietnam, finding 3 security resources, 3 security demands, and 2 personal resources that affected security engagement and security compliance burnout as the mediators of security compliance [25].

#### OB Framework

OB research has been around for about 100 years now [26]. There are 3 levels at which OB can be studied: the individual, the group, and the organizational level [27]. By analyzing information security–related compliance, our study focuses on how resident physicians behave as individuals in the organization and which job-related antecedents promote or inhibit compliance. Amankwa et al [28] chose—among others—concepts from OB research to explain information security–related compliance, showing that supportive organizational culture and end-user involvement had a significant effect on employees’ attitudes toward compliance, which in turn had a positive effect on their behavioral intentions.

### Research Model and Hypotheses

**Figure 1:**
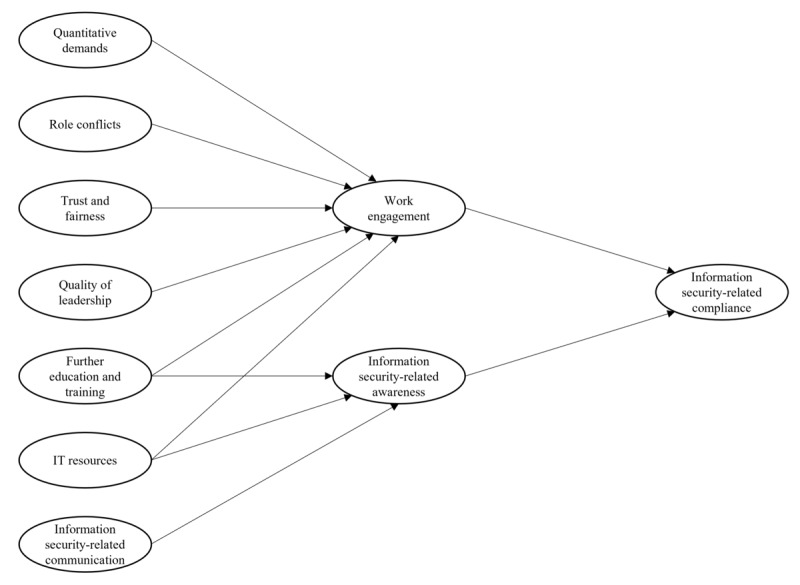
Research model.

Our initial research model is presented in Figure 1. The relevant constructs, definitions, and corresponding items of the questionnaire can be found in Multimedia Appendix 1.

As the first mediator, we have chosen work engagement (“WENG”). In a revised version of the JDRM presented by Schaufeli and Bakker [22], work engagement is considered to be a mediator of the relationship between job resources and job-related outcomes. The authors explained their findings through an underlying motivational process. Next to quality of leadership (“LEAD”), education and training (“EDUC”), and IT resources (“ITRE”), we chose trust and fairness (“TRFA”) as the fourth potential resource. Unlike Schaufeli and Bakker [22], we also assumed a negative effect of certain job demands—quantitative demands (“QUAD”) and role conflicts (“ROLC”)—on work engagement. Here, we wanted to refer to the specific working conditions of physicians in hospitals, which are in general associated with high quantitative demands, but also—as Hedström et al [19] showed—different value conflicts.

Awareness (“AWAR”), as the second mediator, is one of the most commonly investigated variables related to information security–related behavior [29-32]. It is mostly considered to play a mediating role between the exogenous variables and the endogenous compliance variable (“COMP”). In our research model, it also acts as a mediator and reports on physicians’ awareness of the risks, threats, policies and responsibilities related to information security in their hospital. In addition to information security–related communication (“COMM”), for which a relationship with information security–related behavior has already been shown in several studies [16,17,28,32], we added EDUC as well as ITRE as potential exogenous variables in our model. Here, the assumption was that both job resources the provision of adequate IT resources and good education and training could lead to greater information security–related awareness. A summary of our research hypotheses is provided in Table 1.

**Table 1:** Research hypotheses.

#	Hypotheses
H1a	Work engagement is positively related to information security–related compliance.
H1b	Information security–related awareness is positively related to information security–related compliance.
H2a	There is a negative relationship between quantitative demands and information security–related compliance mediated by work engagement.
H2b	There is a negative relationship between role conflicts and information security–related compliance mediated by work engagement.
H2c	There is a positive relationship between trust and fairness and information security–related compliance mediated by work engagement.
H2d	There is a positive relationship between quality of leadership and information security–related compliance mediated by work engagement.
H2e	There is a positive relationship between further education and training and information security–related compliance mediated by work engagement.
H2f	There is a positive relationship between IT resources and information security–related compliance mediated by work engagement.
H3a	There is a positive relationship between further education and training and information security–related compliance mediated by awareness.
H3b	There is a positive relationship between IT resources and information security–related compliance mediated by awareness.
H3c	There is a positive relationship between information security–related communication and information security–related compliance mediated by awareness.

## Methods

### Study Design

We used a cross-sectional, survey-based study design. A structured questionnaire in German was developed and designed with the free online survey tool LimeSurvey, whereby the first page contained information on the target group, the research project, the content of the questionnaire, and the estimated processing time. After accepting the privacy policy, participants were taken to the second page with demographic questions. These were followed by the main part including seven sections: (1) working conditions; (2) resilience; (3) job satisfaction and work engagement; (4) IT resources, information security, and data protection; (5) information security–related awareness and compliance; (6) technical affinity and innovative work behavior; and (7) mobile device usage. At the end of the survey, participants had the opportunity to share their comments with us. The sections relevant to this report are explained below.

- Working conditions: To assess working conditions, we used the following scales from the Copenhagen Psychosocial Questionnaire (COPSOQ): Quantitative Demands, Predictability, Role Conflicts, Quality of Leadership, Social Support, Feedback, Sense of Community, Trust and Fairness, and Appreciation. The COPSOQ is an internationally established instrument to measure psychosocial work factors, with good to very good validity and reliability for most of its scales [33]. In Germany, the third version of the questionnaire, which we used in our study, was published in 2019 [34]. In contrast to the original questionnaire, we divided the Social Support and Feedback scales into 2 subscales each (supervisors versus colleagues) to separate social support and feedback from supervisors from social support and feedback from colleagues, which could be rated differently, especially in hierarchical organizations. In addition, we used 2 scales (Uncertainty, and Further Education and Training) and 2 individual questions on working hours and shifts from the German instrument for stress-related job analysis for hospital physicians (ISAK) [35,36]. We also included 4 self-developed items regarding IT resources in the hospital because we could not find a suitable scale in the research literature.
- Information security–related awareness and compliance: In addition to state-of-the-art technical information security solutions, employees of an organization should be aware of the importance of information security and trained accordingly to behave in a compliant manner [31,37]. The items that we used to assess information security–related communication, awareness, self-efficacy, top management commitment, and compliance are based on the works of Hu et al [15], D’Arcy and Greene [16], Karlsson et al [30], and Solomon and Brown [17]. We adapted the items to the clinical situation. Overall, this resulted in 4 items on communication and awareness, 2 on self-efficacy, 1 on top management commitment, and 4 on compliance.

The Work Engagement scale is also part of the COPSOQ and comprises 3 items. All English scales and single items were translated into German, checked independently by 2 bilinguals, and then adapted based on their comments. We used 5-point Likert scales, spanning from “To a very high degree” to “To a very low degree,” from “Not true” to “Completely true,” from “Always” to “Never/almost never,” and from “Strongly disagree” to “Strongly agree,” respectively.

We followed the Checklist for Reporting Results of Internet E-Surveys (CHERRIES; Multimedia Appendix 2) [38]. To ensure content validity, the survey was reviewed by faculty members and statisticians and modified accordingly. It was then piloted with a group of residents, who highlighted and took notes on any remaining ambiguities, which we corrected in the final questionnaire. We also tested the usability and technical functionality of the questionnaire before fielding it.

Determining the sample size for structural equation modeling (SEM) is difficult due to its flexibility. There are no generalized guidelines regarding sample size requirements [39]. Therefore, we used the rule of thumb of N≥200 [40].

### Data Collection

Data were collected between March and June 2022. Our target group comprised physicians who are currently undergoing medical specialist training/residency training in hospitals in Germany (henceforth referred to as “residents” or “resident physicians”). We focused on residents because they predominantly belong to Generation Y and, compared with older generations, have been used to using digital technologies and especially mobile devices since childhood or youth; therefore, they represent a homogeneous target group in terms of this characteristic. An invitation with a link to the questionnaire was sent directly to the residents by email or social media channels, or indirectly by our contacts in the medical field. Important contacts were chief physicians, senior physicians, university professors, hospital managers, alumni networks, as well as presidents of the German medical societies. In addition, we asked medical experts with significant influence on social media platforms to share the link. The Hartmannbund, an important association of physicians in Germany, forwarded the link to its resident members.

### Data Analysis

In total, we examined 2 job demands (QUAD and ROLC) and 5 job resources (TRFA, LEAD, EDUC, ITRE, and COMM) as exposure variables, 2 mediators (WENG and AWAR), and 1 outcome variable (COMP). All variables were measured using Likert scales and are interval scaled. A summary of our research hypotheses is provided in Table 1. To be statistically precise, we would like to highlight that the hypotheses are always intended to analyze the unique effects of the variables in the model and not the bivariate correlations.

For the analysis of our collected data, we applied SEM using the free software environment for statistical computing and graphics R (R Foundation for Statistical Computing) and the *lavaan* package. SEM refers to a family of data analysis techniques for complex relationships between multiple variables in a single research model and has become popular in the social and behavioral sciences [41,42]. Based on our research model and hypotheses, we followed the widely adopted 2-step approach to SEM by Anderson and Gerbing [43], in which the assessment of the measurement model is followed by the analysis of the structural model including tests of the research hypotheses. While the measurement model describes how the latent variables are measured by the indicator variables, the structural model shows how the latent variables are related. In the first step, a confirmatory factor analysis was performed to determine whether the initially proposed measurement model fits the data or needs to be respecified accordingly. Various fit indices exist for evaluating the global model fit. In accordance with Hu and Bentler [44], we used the standardized root mean squared residual (SRMR), the comparative fit index (CFI), and the root mean squared error of approximation (RMSEA). The authors recommended the following thresholds for fit indices that characterize a good global fit: SRMR<.08, CFI≥.95, and RMSEA<.06 [44]. Modification indices were also analyzed to determine the local fit. Here, we only made changes that were theoretically sensible in terms of our model. In addition, the results obtained were assessed regarding reliability, validity, and plausibility. If respecification was indicated, the entire evaluation process was repeated until a good model fit was achieved. In the second step, the relationships between the latent variables are analyzed by again evaluating the model fit and afterward interpreting the path coefficients β and the coefficient of determination *R*². To calculate the results, we used the robust WLSMV (weighted least squares means and variance adjusted) estimator. To ensure model identification, we only used factors with 3 or more items in the initial measurement model.

### Ethical Considerations

Ethical review and approval were waived for this study. We are thus following the guidelines of the German Research Foundation (DFG) and the German Data Forum (RatSWD). The RatSWD is an independent body of empirically working scientists and representatives of the most important public institutions for data collection in Germany. It recommends a careful self-examination of research ethics in order to decide whether an ethical review by a committee is necessary for the planned research project. After careful self-examination, we were able to classify our research project as unobjectionable in terms of research ethics: neither patients nor other vulnerable groups took part in the study. Also, our study was not likely to trigger powerful emotions or cause severe psychological stress or traumatic experiences. It did not involve physical risks to the participants or result in physical pain. Furthermore, the participants were informed of the study. Potential risks of participation (such as social risks, risks of criminal or civil liability, financial loss, professional disadvantages, or damage to reputation; risk due to a difficult security situation in the study room) did not exist. Participation in the study did not involve any type of deception [45,46]. No compensation was provided.

## Results

### Sample Characteristics

A total of 611 people entered the survey, of whom 349 completed it. An exact statement on the response rate cannot be made because we do not have information on the number of residents who received the questionnaire indirectly through our contacts. Data from 6 participants had to be excluded due to conspicuous response patterns (n=4), work in a hospital abroad (n=1), and specific information in the comment section (n=1). Furthermore, only data from participating physicians who had a clear understanding of information security as distinct from data protection were included. This was checked with the help of a single comprehension item. Overall, data from 281 participants could be used for our analyses.

Table 2 shows the sociodemographic characteristics of the study participants. A large majority of the participants were female (173/281, 61.6%). The two age groups with the highest frequency were 31-35 years (114/281, 40.6%) and 26-30 years (108/281, 38.4%). There was a total of 16 specialties represented by at least two participants. Most participants were part of a residency program for internal medicine (62/281, 22.1%), followed by anesthesiology (47/281, 16.7%), surgery (43/281, 15.3%), and pediatrics and adolescent medicine (30/281, 10.7%). Participants were distributed across all residency levels, with the fewest residents in their fourth year (43/281, 15.3%) and most residents in their fifth year or above (82/281, 29.2%). The majority of the participants worked in a public hospital (174/281, 61.9%), while approximately one-fifth (62/281, 22.1%) worked in a nonprofit hospital and 14.2% (40/281) in a private hospital. Most of the participants were employed in a university/teaching hospital (230/281, 81.9%). The size of the hospitals (measured by the number of beds) in which the residents underwent their training varied, whereby most of them worked in a hospital with more than 800 beds (109/281, 38.8%), followed by hospitals with 300-800 beds (104/281, 37%). Fewer than one-fifth (52/281, 18.5%) of the residents had a job in a hospital with less than 300 beds.

**Table 2:** Sample characteristics (n=281).

Variable			Value, n (%)
**Gender**			
	Female	173 (61.6)	
	Male	108 (38.4)	
	Other	0 (0)	
**Age group (years)**			
	21-25	7 (2.5)	
	26-30	108 (38.4)	
	31-35	114 (40.6)	
	36-40	30 (10.7)	
	40 or older	22 (7.8)	
**Specialty**			
	Internal medicine	62 (22.1)	
	Anesthesiology	47 (16.7)	
	Surgery	43 (15.3)	
	Pediatrics and adolescent medicine	30 (10.7)	
	Other	99 (35.2)	
**Residency level**			
	First year	59 (21)	
	Second year	47 (16.7)	
	Third year	50 (17.8)	
	Fourth year	43 (15.3)	
	Fifth year or higher	82 (29.2)	
**Hospital sponsorship**			
	Private	40 (14.2)	
	Public	174 (61.9)	
	Nonprofit	62 (22.1)	
	I don’t know	5 (1.8)	
**University or teaching hospital**			
	Yes	230 (81.9)	
	No	48 (17.1)	
	I don’t know	3 (1.1)	
**Hospital size (beds)**			
	Fewer than 300	52 (18.5)	
	300-800	104 (37)	
	More than 800	109 (38.8)	
	I don’t know	16 (5.7)	

### Evaluation of the Measurement Model

We analyzed the model fit, the reliability, and the validity of the measurement model and respecified it accordingly until we received sufficient results. We began with an assessment of the global fit of our original measurement model. Table 3 shows the measurement model fit indices for the initial and the final models. It revealed that the CFI was below the recommended threshold, whereas the RMSEA and SRMR already showed acceptable values.

**Table 3:** Fit indices of the measurement model (N=281).

Model	Chi-square (*df*)	*P* value	CFI^a^	RMSEA^b^	SRMR^c^
Initial measurement model	1217.7 (657)	<.001	.933	.055	.070
Respecified final measurement model	794.3 (514)	<.001	.965	.044	.055
Recommended thresholds	—^d^	≥.05	≥.95	<.06	<.08

^a^CFI: comparative fit index.

^b^RMSEA: root mean squared error of approximation.

^c^SRMR: standardized root mean squared residual.

^d^Not applicable.

Looking at Cronbach α of the latent variables of the initial measurement model, we received a poor value (<0.70) for the latent variable AWAR. Here, we decided to drop the first two items (AWAR1 and AWAR2) because, compared to the third and fourth items, they seem to be too special and an own latent variable, asking about concrete information security–related knowledge. One reason could be that at the time of the survey, information security policies and officers were not yet implemented in most hospitals and could, therefore, not be known to the physicians. After removing those 2 items, the Spearman-Brown coefficient was acceptable.

We further respecified the model according to the modification indices and added an error covariance between QUAD1 and QUAD2. We then dropped items with loadings <.50 (TRFA1 and ITRE1). Although the *χ*^2^ goodness-of-fit statistic was still significant (*P*<.001), these respecifications resulted in global fit indices above the thresholds, as shown in Table 3, meaning that the measurement model can be considered to be an acceptable approximation of the empirical data. According to the Fornell-Larcker [47] criterion, we also compared the square root of the average variance extracted (AVE) for each variable with the corresponding correlations of all other constructs [47]. Since the square root of the AVE was higher than the correlations on the respective horizontal and vertical lines, as shown in Table 4, the discriminant validity of the variables can be confirmed. In addition, the AVE of each variable should be higher than .50 to achieve acceptable convergent validity. All AVEs were above this threshold. Afterward, we looked again at Cronbach α and the composite reliability of each latent variable. All values were higher than the recommended threshold of .70, indicating acceptable reliability of the latent variables. In summary, we obtained acceptable model fit, reliability, and validity for the measurement model. A summary of the quality indicators of the measurement model is presented in Table 5.

**Table 4:** Mean, SD, and correlations after item exclusion (N=281).

Latent variable		Mean (SD)	1	2	3	4	5	6	7	8	9	10
**1. QUAD**		3.672 (0.688)										
	r		**.778^a^**									
	*P* value											
**2. ROLC**		3.172 (0.781)										
	r		.422	**.746**								
	*P* value		<.001									
**3. TRFA**		2.961 (0.688)										
	r		-.225	-.393	**.732**							
	*P* value		<.001	<.001								
**4. LEAD**		2.897 (0.857)										
	r		-.341	-.391	.348	**.822**						
	*P* value		<.001	<.001	<.001							
**5. EDUC**		2.898 (0.897)										
	r		-.389	-.463	.300	.627	**.820**					
	*P* value		<.001	<.001	<.001	<.001						
**6. ITRE**		2.367 (0.910)										
	r		-.166	-.231	.398	.138	.141	**.813**				
	*P* value		.002	<.001	<.001	.013	.005					
**7. COMM**		2.686 (0.901)										
	r		-.155	-.199	.313	.173	.254	.350	**.792**			
	*P* value		.005	<.001	<.001	.001	<.001	<.001				
**8. WENG**		3.471 (0.674)										
	r		-.135	-.261	.184	.379	.277	.086	.118	**.808**		
	*P* value		.033	<.001	.001	<.001	<.001	.226	.027			
**9. AWAR**		3.835 (0.971)										
	r		-.087	-.076	.076	.188	.182	-.004	.256	.121	**.904**	
	*P* value		.141	.244	.197	.002	.001	.797	<.001	.036		
**10. COMP**		3.563 (0.731)										
	r		-.080	-.196	.175	.201	.239	.084	.337	.219	.502	**.776**
	*P* value		.234	<.001	.002	<.001	<.001	.129	<.001	<.001	<.001	

^a^Bold values on the diagonal are the square root of the corresponding average variance extracted (AVE).

**Table 5:** Measurement model quality indicators (n=281).

Latent variable		Loadings	Cronbach α	Composite reliability	AVE^a^
**Quantitative demands**			0.854	.883	.605
	QUAD1	.644	—^b^	—	—
	QUAD2	.710	—	—	—
	QUAD3	.899	—	—	—
	QUAD4	.888	—	—	—
	QUAD5	.715	—	—	—
**Role conflicts**			0.743	.789	.557
	ROLC1	.827	—	—	—
	ROLC2	.711	—	—	—
	ROLC3	.693	—	—	—
**Trust and fairness**			0.720	.774	.536
	TRFA1	Dropped	—	—	—
	TRFA2	.689	—	—	—
	TRFA3	.835	—	—	—
	TRFA4	.661	—	—	—
**Quality of leadership**			0.861	.892	.676
	LEAD1	.884	—	—	—
	LEAD2	.872	—	—	—
	LEAD3	.806	—	—	—
	LEAD4	.716	—	—	—
**Further education and training**			0.853	.891	.672
	EDUC1	.784	—	—	—
	EDUC2	.910	—	—	—
	EDUC3	.721	—	—	—
	EDUC4	.852	—	—	—
**IT resources**			0.776	.851	.661
	ITRE1	Dropped	—	—	—
	ITRE2	.831	—	—	—
	ITRE3	.934	—	—	—
	ITRE4	.647	—	—	—
**Information security–related** **communication**			0.826	.870	.627
	COMM1	.761	—	—	—
	COMM2	.731	—	—	—
	COMM3	.788	—	—	—
	COMM4	.880	—	—	—
**Work engagement**			0.777	.846	.652
	WENG1	.658	—	—	—
	WENG2	.969	—	—	—
	WENG3	.765	—	—	—
**Information security–related awareness**			0.855^c^	.899	.817
	AWAR1	Dropped	—	—	—
	AWAR2	Dropped	—	—	—
	AWAR3	.866	—	—	—
	AWAR4	.940	—	—	—
**Information security–related compliance**			0.789	.853	.602
	COMP1	.896	—	—	—
	COMP2	.801	—	—	—
	COMP3	.842	—	—	—
	COMP4	.504	—	—	—

^a^AVE: average variance extracted.

^b^Not applicable.

^c^Spearman-Brown coefficient.

### Analysis of the Structural Model and Hypotheses Testing

**Figure 2:**
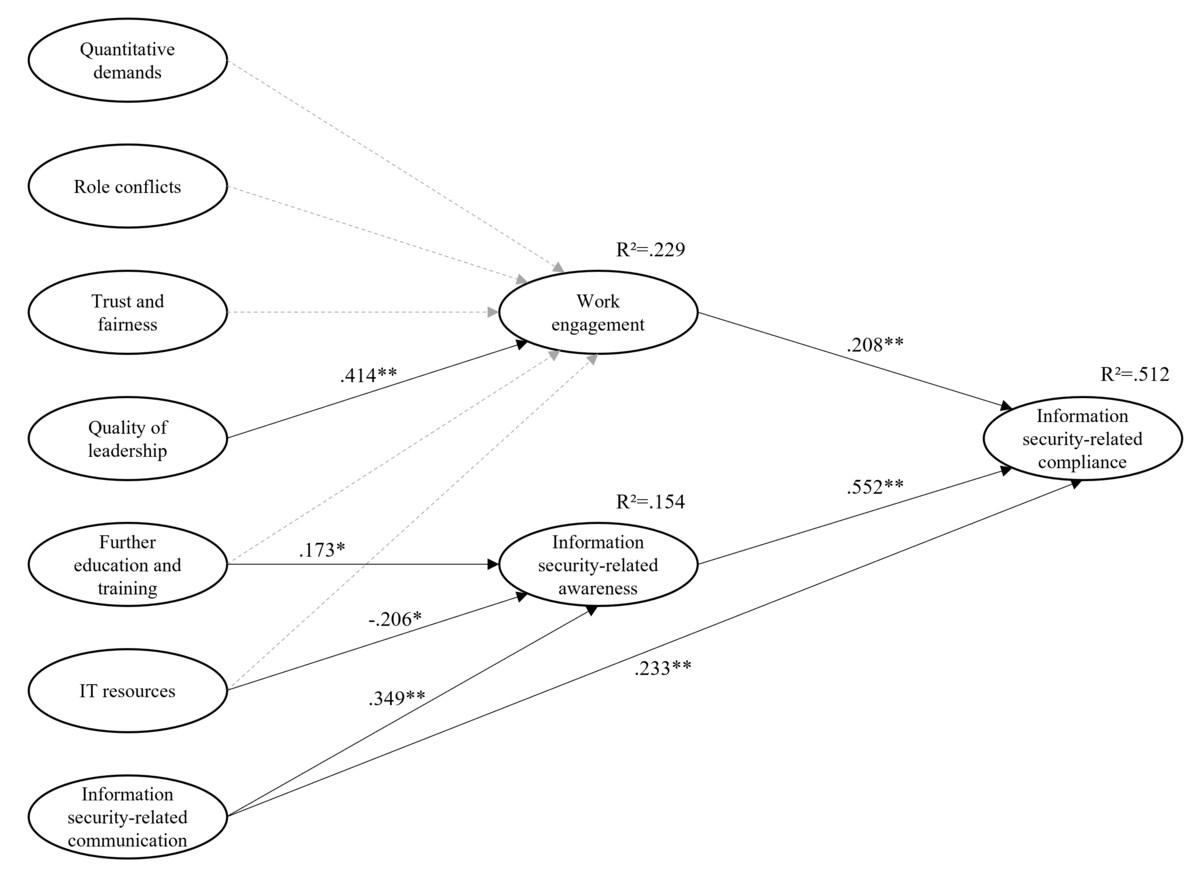
Structural model and standardized direct effects. *P<.05, **P<.01. Dashed gray arrows indicate statistically nonsignificant effects.

The initial structural model already showed a good global fit (Table 6). Nevertheless, we followed a proposed modification index and added a direct effect of COMM on COMP, resulting in the final structural model. The *R*² value for the endogenous variable COMP was .512, indicating that 51.2% of the variance of this variable can be explained by our structural model (Figure 2). The *R*² values for the mediators were .229 (WENG) and .154 (AWAR).

Figure 2 also presents the significant direct effects and standardized path coefficients (β). A summary of the hypotheses tested is provided in Multimedia Appendix 3. The results show a significant relationship between LEAD and WENG (β=.414*, P*=.001). There were no significant unique effects of the other exogenous variables tested (QUAD, ROLC, TRFA, EDUC, and ITRE) on WENG (all *P*>.05). Furthermore, we found a significant positive relationship between EDUC and AWAR (β=.173*, P*=.01), as well as between COMM and AWAR (β=.349*, P*<.001). In addition, there was a significant negative relationship between ITRE and AWAR (β=–.206*, P*=.01). Both mediators WENG (β=.208*, P*=.001) and AWAR (β=.552*, P*<.001) were significantly associated with the endogenous variable COMP, which supports H1a and H1b.

In order to test the mediation hypotheses, we calculated the partial indirect effects. A summary of the results is also presented in Multimedia Appendix 3. The results showed that WENG significantly mediated the relationship between LEAD and COMP (*P*=.03), supporting hypothesis H2d. The positive indirect effect resulted from the positive direct effects between LEAD and WENG and between WENG and COMP. AWAR mediated the relationships between the exogenous variables EDUC (*P*=.02), ITRE (*P*= .02), and COMM (*P*<.001) and the endogenous variable COMP, which supports H3a and H3c. Since we expected a positive indirect effect of ITRE on COMP, H3b could not be confirmed. The positive indirect effects of EDUC and COMM resulted from the positive direct effects involved.

**Table 6:** Fit indices of the structural model (n=281).

Model	Chi-square (*df*)	*P* value	CFI^a^	RMSEA^b^	SRMR^c^
Initial structural model	799.1 (527)	<.001	.966	.043	.059
Respecified final structural model	769.5 (526)	<.001	.970	.041	.056
Recommended thresholds	—^d^	≥.05	≥.95	<.06	<.08

^a^CFI: comparative fit index.

^b^RMSEA: root mean squared error of approximation.

^c^SRMR: standardized root mean squared residual.

^d^Not applicable.

## Discussion

### Principal Findings and Comparison With Previous Work

With this study, we could statistically prove the influence of resident physicians’ job resources and job demands on their information security–related compliance through the mediating roles of work engagement and awareness. This is one of the first studies to empirically analyze organizational antecedents of physicians’ information security–related behavior in hospitals. Intensified research activities in this field are of great importance given that increasing digitization in hospitals and the associated risks from cybersecurity attacks require—in addition to technical solutions—well-considered organizational measures to enhance information security.

It was shown from the results that the information security–related compliance of resident physicians is significantly related to their work engagement. The assumed positive effect from our hypothesis was, therefore, confirmed. Pham et al [25] already described this relationship in their explorative study using interview analyses, which could be now supported by our results. In line with the findings of the study by Schaufeli and Bakker [22] and their revised version of the JDRM, in which they stated that “engagement is exclusively predicted by available job resources,” we did not find unique relationships between the job demands involved (quantitative demands and role conflicts) and work engagement. Both demands also had no significant indirect effect on compliance. It can be, therefore, assumed that decreasing job demands would not be the most effective strategy in enhancing physicians’ information security–related compliance, which would need to be further investigated by subsequent studies. Instead, our study highlights the importance of the job resource “quality of leadership,” which had a significant indirect effect on information security–related compliance mediated by work engagement. Based on that, we can conclude that supervisors have the power to increase resident physicians’ compliance through the motivational path: a good relationship with their direct supervisors, who are mostly senior physicians, increases resident physicians’ work engagement, which leads to better compliance with the hospital's information security measures. According to our study, this relationship can be supported by the supervisor’s efforts in the areas of staff development, job satisfaction, work planning, and conflict-solving. In light of the increasing digitization, this should be accompanied by digital and change management competencies [48].

Besides work engagement, awareness was the second mediator in our model, and based on our results, it is also significantly related to information security compliance. Here, our findings are in line with the results of several other studies, analyzing the role of awareness as a mediator in various industries [22,31,32]. We found 2 job resources with a unique positive effect on awareness and also a significant indirect effect on compliance: information security–related communication and further education and training. Communication is a highly effective factor in improving information security–related compliance, as has been shown in other studies [16,17]. Regular, comprehensible information security–related communication increases physicians’ awareness of potential information security risks and threats. Here, we see a particular responsibility on the part of the hospital management. A communication strategy should be developed that makes all employees aware of the risks and threats and their particular information security responsibilities. In addition, information security–related communication had a direct effect on compliance. It could be assumed here that good communication also triggers automatic mechanisms for resolving critical situations. Furthermore, our results show that awareness can be created not only by communication but also by good education and training. Modern training concepts take a holistic approach and use various methods to raise awareness, for example, through personalized phishing simulations for training purposes and classic learning sessions with subsequent skills tests and personalized follow-up training based on individual results. Contrary to our hypothesis, IT resources are negatively related to awareness. One possible explanation could be that digitization and technologization increase confidence in the security of the tools used. On the other hand, if only outdated IT resources are available, physicians might be more sensitive to possible security risks.

Overall, our study provides initial insights into how the hospital as an organization could influence the individual information security–related behavior of resident physicians during clinical practice. In our opinion, the core element is the creation of an information security culture as a subculture of the organizational culture, which increases both the work engagement and the awareness of resident physicians through good leadership, information, and training.

### Limitations

A few limitations should be taken into account when considering and evaluating our results, one of which pertains to the study design. First, since our study was conducted in Germany, it is not possible to directly apply the results to other countries. However, our study is intended to create incentives to conduct studies in hospitals in other countries to explore organizational factors for improving information security–related behavior. Second, when using a survey, comprehension problems on the part of the participants cannot be identified and addressed. However, we believe that the approach was the most suitable for the aim of the study and the selected target group as—for example—interviews would have resulted in a much smaller sample, which might have reduced the validity of the results. Third, the cross-sectional design does not allow us to form causal relationships between organizational antecedents, work engagement, awareness, and compliance, but only correlations. Any alterations in physicians’ behavior due to changes in organizational factors can only be assessed with a longitudinal design. Nevertheless, we assume causal relationships based on logical considerations.

Even though it was an anonymous questionnaire, the risk of social desirability bias remains, with participants trying to be much more positive about their job-related attitudes and behavior. Furthermore, the fact that we cannot precisely determine the response rate represents another limitation. Since we do not have information on the number of residents who received the questionnaire indirectly through our contacts, for example, through chief physicians or the hospital management, we are not able to make an exact statement on the number of residents being invited, which is the basis for calculating the response rate. However, based on the information available to us, we estimate that fewer than 10% of those who received the questionnaire actually responded. If there are systematic differences between the responders and nonresponders, the results of our survey may not be representative of the target population. This so-called nonresponse bias may threaten the external validity of our study by reducing the representativeness of the results. However, previous research suggests that physician surveys are less susceptible to nonresponse bias than general population studies because they are a more homogeneous study population [49]. Furthermore, it is not always the case that a low response rate automatically reduces the representativeness, which is why the response rate should not be considered in isolation [50]. We believe the main reason for the low response rate was the heavy workload, which did not allow physicians the time to participate. Another indicator of the representativeness of a study is the sampling method [51]. The approach chosen for the data collection, in which residents were invited through different channels, may have led to a selection bias. For example, it is possible that digitally active and networked physicians were primarily addressed. The high proportion of physicians who received the questionnaire through the Hartmannbund may also contribute to the selection bias. Our study may have appealed to physicians with a higher average digital affinity than in the target population. To sum up, statistical conclusions on the entire target population should, therefore, always be drawn, taking into account the supposedly limited representativeness.

Future studies could use a longitudinal design to investigate the specific introduction of information security–related measures and the associated change in behavior. Furthermore, objective criteria, such as the number of breaches, could also be used as a measure of compliance instead of subjective self-reports. In addition, qualitative studies in the form of interviews are suitable for recording specific activities, opinions, perceptions, and concerns of physicians. It would also be interesting to conduct a study among senior physicians who have been working in hospitals for significantly longer and may need other measures to ensure that they behave in accordance with information security. For example, existing habits may be more difficult to change by adjusting resources and demands, and the resource “quality of leadership” may play a weaker role than for resident physicians.

### Conclusions

A high standard of information security can only be achieved holistically through coordinated technical and organizational measures. In hospitals, this includes achieving a great level of work engagement among physicians through good leadership, but also creating awareness of the risks and threats relating to information security through communication and training. Hospital management is required to establish an information security culture that is informative and motivating and that raises awareness.

## Appendix

Multimedia Appendix 1 Constructs, definitions, and corresponding items of the questionnaire.

Multimedia Appendix 2 Checklist for Reporting Results of Internet E-Surveys (CHERRIES).

Multimedia Appendix 3 Summary of the hypotheses tested.

## Abbreviations

AVE: average variance extracted
BYOD: bring your own device
CFI: comparative fit index
CHERRIES: Checklist for Reporting Results of Internet E-Surveys
COPSOQ: Copenhagen Psychosocial Questionnaire
GDPR: General Data Protection Regulation
JDRM: Job Demands-Resources Model
OB: Organizational Behavior
RMSEA: root mean squared error of approximation
SEM: structural equation modeling
SRMR: standardized root mean squared residual
TPB: Theory of Planned Behavior
WLSMV: weighted least squares means and variance adjusted

## Data Availability

The datasets used and/or analyzed in the context of this study are available from the corresponding author upon reasonable request.
