# Supplementary material for: The Role of the Organization in Promoting Information Security–Related Behavior Among Resident Physicians in Hospitals in Germany: Cross-Sectional Questionnaire Study
Source: J Med Internet Res. 2025 Jan 7;27:e46257. doi: 10.2196/46257 (PMC11751644; doi:10.2196/46257)
Supplement: Multimedia Appendix 1 [file jmir_v27i1e46257_app1.docx]

Multimedia Appendix 1.

| **Construct** |  |
| --- | --- |
|  |  |
| **Quantitative demands** |  |
| Definition | “Quantitative Demands deal with how much one has to achieve in ones work. Quantitative Demands can be assessed as an incongruity between the amount of tasks and the time available to perform these tasks in a satisfactory manner.” [33] |
| Items |  |
| QUAD1 | Do you have to work very fast? |
| QUAD2 | Do you work at a high pace throughout the day? |
| QUAD3 | How often do you not have time to complete all your work tasks? |
| QUAD4 | Do you get behind with your work? |
| QUAD5 | Do you have to do overtime? |
|  |  |
| **Role conflicts** |  |
| Definition | “Role Conflicts stem from two sources. The first source is about possible inherent conflicting demands within a specific task. The second source is about possible conflicts when prioritizing different tasks.” [33] |
| Items |  |
| ROLC1 | Are contradictory demands placed on you at work? |
| ROLC2 | Do you sometimes have to do things, which ought to have been done in a different way? |
| ROLC3 | Do you sometimes have to do things, which seem to you to be unnecessary? |
|  |  |
| **Trust and fairness** |  |
| Definition | “Vertical Trust deals with whether the employees can trust the management and vice versa. Vertical Trust can be observed in the communication between the management and the employees.” [33]  “Justice and respect in the workplace is about if workers are treated fairly.” [33] |
| Items |  |
| TRFA1 | Does the management trust the employees to do their work well? |
| TRFA2 | Can the employees trust the information that comes from the management? |
| TRFA3 | Are conflicts resolved in a fair way? |
| TRFA4 | Is the work distributed fairly? |
|  |  |
| **Quality of leadership** |  |
| Definition | “Quality of Leadership deals with the next higher managers' leadership in different contexts and domains.” [33] |
| Items |  |
|  | To what extent would you say that your immediate superior… |
| LEAD1 | …makes sure that the members of staff have good development opportunities? |
| LEAD2 | …gives high priority to job satisfaction? |
| LEAD3 | …is good at work planning? |
| LEAD4 | …is good at solving conflicts? |
|  |  |
| **Further education and training** |  |
| Definition | Further education and training describes the opportunities for professional development in the department. (own definition) |
| Items |  |
| EDUC1 | In our department, inexperienced medical colleagues have ample opportunity to benefit from the knowledge and skills of experienced colleagues. |
| EDUC2 | Specialist training for resident physicians is well supported in our department. |
| EDUC3 | New physicians are well trained in our department. |
| EDUC4 | Further education and training is well promoted in our department. |
|  |  |
| **IT resources** |  |
| Definition | IT resources describes the support of the physicians by the IT equipment and IT staff. (own definition) |
| Items |  |
| ITRE1 | WiFi is available throughout your hospital for internal use. |
| ITRE2 | The IT equipment in your hospital is outdated. |
| ITRE3 | The IT equipment in your hospital limits you in your daily work. |
| ITRE4 | IT issues are resolved immediately by employees in the IT department. |
|  |  |
| **Information security-related communication** |  |
| Definition | “The organisation’s continuous education and communication of the importance of information security to their employees in an effort to ensure compliance behaviour.”[17] |
| Items |  |
| COMM1 | The hospital sensitizes employees to the topic of information security. |
| COMM2 | The hospital provides adequate information security training. |
| COMM3 | The employees at the hospital have a clear understanding of their information security responsibilities. |
| COMM4 | The hospital’s information security policies are strongly enforced. |
|  |  |
| **Work engagement** |  |
| Definition | “This dimension deals with the attachment you feel to the task independently of how you experience your workplace.” [33] |
| Items |  |
| WENG1 | At my work, I am full of energy. |
| WENG2 | I am enthusiastic about my job. |
| WENG3 | I am immersed in my work. |
|  |  |
| **Information security-related awareness** |  |
| Definition | “[Information security-related awareness] reports respondents’ awareness of the information security regulations in their organisation.” [30] |
| Items |  |
| AWAR1 | I am aware of the information security policies at the hospital. |
| AWAR2 | I know the responsible information security officer at the hospital. |
| AWAR3 | I understand the risks of lacking information security. |
| AWAR4 | I am aware of the potential threats to information security at the hospital. |
|  |  |
| **Information security-related compliance** |  |
| Definition | Information security-related compliance describes the physician’s belief that he or she complies / will comply with the hospital’s information security policy. (own definition) |
| Items |  |
| COMP1 | I do my best to follow the hospital's rules on information security. |
| COMP2 | I am sure that I do my work in accordance with the hospital's rules on information security. |
| COMP3 | I intend to comply with the hospital's rules on information security in the future. |
| COMP4 | I intend to read or attend required information security training courses in the future. |
