# Supplementary material for: The Role of the Organization in Promoting Information Security–Related Behavior Among Resident Physicians in Hospitals in Germany: Cross-Sectional Questionnaire Study
Source: J Med Internet Res. 2025 Jan 7;27:e46257. doi: 10.2196/46257 (PMC11751644; doi:10.2196/46257)
Supplement: Multimedia Appendix 3 [file jmir_v27i1e46257_app3.docx]

Multimedia Appendix 3.

| **#** | **Hypotheses** | **Standard. effect** | ***p*** | **Supported / Not supported** |
| --- | --- | --- | --- | --- |
|  |  |  |  |  |
| H1a | (+) WENG → COMP | .208 | .001 | Supported |
| H1b | (+) AWAR → COMP | .552 | < .001 | Supported |
|  |  |  |  |  |
| H2a | (-) QUAD → WENG → COMP | .029 | .131 | Not supported |
| H2b | (-) ROLC → WENG → COMP | -.039 | .116 | Not supported |
| H2c | (+) TRFA → WENG → COMP | 0 | .993 | Not supported |
| H2d | (+) LEAD → WENG → COMP | .086 | .027 | Supported |
| H2e | (+) EDUC → WENG → COMP | 0 | .991 | Not supported |
| H2f | (+) ITRE → WENG → COMP | -.001 | .944 | Not supported |
|  |  |  |  |  |
| H3a | (+) EDUC → AWAR → COMP | .096 | .015 | Supported |
| H3b | (+) ITRE → AWAR → COMP | -.114 | .018 | Not supported* |
| H3c | (+) COMM → AWAR → COMP | .192 | < .001 | Supported |

Note.(-): Negative effect was expected. (+): Positive effect was expected.
*We expected a positive indirect effect, which could not be confirmed.
